# Assessing pediatricians’ readiness for artificial intelligence: a cross-sectional study in Istanbul, Türki̇ye

**DOI:** 10.1186/s12887-026-07088-8

**Published:** 2026-06-03

**Authors:** Eren Güzeloğlu, Belen Ateş, Feyza Aydin Özgür, Sevi̇lay Kök, Hüseyi̇n Dağ, Vefi̇k Arica

**Affiliations:** Department of Pediatrics, University of Health Sciences, Prof. Dr. Cemil Taşcıoğlu City Hospital, Istanbul, Türkiye

**Keywords:** Artificial Intelligence, Pediatrics, Health services, Child

## Abstract

**Background:**

Artificial intelligence (AI) is increasingly integrated into healthcare, including pediatrics, offering new opportunities for diagnosis, management, and decision support. However, the effective implementation of AI depends largely on healthcare professionals’ knowledge, attitudes, and readiness to adopt these technologies. This study aimed to evaluate pediatricians’ general attitudes toward artificial intelligence and their level of readiness for medical AI in Istanbul, Türkiye at tertiary hospital. Distinct from previous research focusing primarily on medical students, this study investigates the AI readiness of practicing pediatricians within a major tertiary hospital in Istanbul, addressing a critical gap in professional clinical workforce assessment.

**Methods:**

This descriptive cross-sectional study was conducted between August 1 and November 1, 2025, at Prof. Dr. Cemil Taşcıoğlu City Hospital. A total of 130 pediatricians participated. Data were collected using a sociodemographic questionnaire, the GAAIS (General Attitudes toward Artificial Intelligence Scale), and the MAIRS-MS (Medical Artificial Intelligence Readiness Scale for Medical Students). The validity and reliability of scales in Turkish have been previously investigated in various studies. The internal consistency of the scales was excellent, with a Cronbach’s alpha coefficient of 0.972 in our study. Statistical analyses were performed using SPSS 27. Correlation, t-test, ANOVA, and non-parametric tests were applied where appropriate.

**Results:**

The mean age of participants was 37.47 ± 9.31 years, and 58.5% were female. The mean AI positive attitude score was 3.81 ± 0.74, and the negative attitude score was 2.83 ± 0.81. The total MAIRS-MS score was 74.97 ± 13.01, indicating a moderate-to-high level of AI readiness. Age was negatively correlated with AI positive attitude and total MAIRS score (*p* < 0.05). Participants with ≥ 21 years of professional experience had significantly lower AI readiness and positive attitude scores than younger participants (*p* < 0.05). A strong positive correlation was found between AI positive attitude and total MAIRS-MS score (*r* = 0.764, *p* < 0.01). Multiple regression analysis revealed that “Ability” (β = 0.516, *p* < 0.001) and “Cognition” (β = 0.323, *p* < 0.001) factors were the strongest predictors of a positive attitude toward AI (R^2^ = 0.658). A strong positive correlation was found between AI positive attitude and total MAIRS-MS score (*r* = 0.764, *p* < 0.01). These results suggest that clinical AI implementation must be accompanied by tailored institutional support and age-specific training to bridge the generational digital divide in pediatric care.

**Conclusion:**

Pediatricians generally demonstrated positive attitudes and moderate-to-high readiness for artificial intelligence in our study. However, advanced age and longer professional experience were associated with lower readiness levels. These findings highlight the need for a structured AI education and training program, especially targeting senior physicians, to ensure effective and ethical integration of AI into pediatric practice.

**Supplementary Information:**

The online version contains supplementary material available at https://doi.org/10.1186/s12887-026-07088-8.

## Introduction

Artificial intelligence (AI) has the potential to transform modern healthcare, from radiology to personalized treatment. However, while these technological advancements are rapidly gaining acceptance in general medicine, the field of pediatrics requires a different approach due to its unique ethical dynamics, growth and development processes, and data sensitivity [1–3].

The potential of AI to reshape modern medicine is particularly significant in pediatrics, facilitating a shift from reactive treatment to proactive well-being management [4–6]. Recent global studies have shown that while pediatricians recognize the potential of AI in areas like growth monitoring and vaccination tracking, there is a notable variation in their readiness to integrate these tools into clinical practice [3, 5, 7]. For instance, research conducted in different healthcare settings has indicated that although healthcare providers maintain a positive outlook on AI’s diagnostic capabilities, they often report a lack of formal training and technical confidence. These findings highlight that the successful adoption of AI is not merely a technical issue but is closely linked to professional acceptance and perceived ease of use [7, 8].

Despite its potential, the effective implementation of AI in pediatric care is often hindered by a lack of understanding regarding its risks and benefits. “Readiness” in this context refers to the extent to which healthcare professionals are mentally and cognitively equipped to adopt these technologies. Existing literature has identified several key predictors of AI readiness, including digital literacy, age, and previous exposure to technology during medical education [6, 8, 9]. Specifically, factors such as “Cognition” the theoretical understanding of AI concepts and “Ability” the perceived proficiency in using AI tools have been found to be critical in determining whether a physician will adopt or resist these emerging technologies [9, 10]. Crucially, AI must be viewed as a tool to complement, rather than replace, the expertise and empathy of physicians [10, 11]. Turkey has identified digitalization in healthcare as a strategic goal in line with its ‘2021–2025 National Artificial Intelligence Strategy’. The success of this national vision depends not only on the technological infrastructure but also on the readiness of the clinical workforce that will implement these Technologies [12, 13].

In daily clinical practice, pediatricians are increasingly expected to engage with artificial intelligence (AI)-supported systems across a wide range of applications, including diagnostic decision support, risk stratification, growth and developmental monitoring, and workflow optimization [3, 7, 10]. However, the integration of AI into pediatric care differs fundamentally from other medical specialties due to the unique vulnerability of children, the need for age- and development-specific clinical interpretation, and the essential involvement of parents or caregivers in medical decision-making processes [3, 5, 7].

Despite its promising potential, the implementation of AI in pediatric settings is accompanied by several clinical, ethical, and organizational challenges. Pediatricians frequently report uncertainty regarding medico-legal responsibility when AI-assisted decisions influence patient outcomes, particularly in the absence of clearly defined regulatory frameworks [8, 11]. Concerns related to data privacy and informed consent are especially pronounced in pediatric populations, where the management of sensitive health data involves minors and guardians [3, 7]. In addition, questions about the reliability and accuracy of AI algorithms in age-dependent and developmentally variable conditions contribute to hesitancy in clinical adoption [7, 8].

Furthermore, there is an ongoing concern that AI technologies may undermine clinical autonomy, professional judgment, and the physician–family relationship, which remains central to pediatric care [1, 3, 5]. Limited access to structured training programs, insufficient institutional support, and a lack of practical guidance for integrating AI into routine workflows further complicate the adoption process [8, 11]. These multifaceted challenges highlight the importance of evaluating pediatricians’ readiness for AI, as successful implementation depends not only on technological advancements but also on clinicians’ knowledge, trust, and preparedness to use these systems safely and effectively in real-world pediatric practice.

A literature review reveals numerous studies examining medical students’ attitudes towards artificial intelligence. However, the preparedness levels of pediatric specialists, who play a central role in clinical decision-making processes and will apply these technologies directly at the patient’s bedside, have been largely overlooked. In particular, the readiness of pediatricians in tertiary hospitals in Türkiye, which operate under heavy patient loads, remains a critical gap in the literature regarding this transformation.

Our study aimed to measure pediatricians’ general attitude towards artificial intelligence and their level of readiness for medical artificial intelligence. While AI’s role in healthcare expands, a significant gap exists in understanding the readiness of practicing pediatricians, as most research focuses on medical students. This study addresses this void within the Turkish healthcare context, specifically investigating how factors such as advanced age, professional experience, and technological familiarity shape AI adoption. By identifying the barriers and facilitators among specialized clinicians in a major Istanbul hospital, we aim to provide essential insights for developing targeted medical education and policy frameworks to prepare the pediatric workforce for the digital transformation of medicine.

## Material-method

### Study design and sampling

The study was conducted at Prof. Dr. Cemil Taşcıoğlu City Hospital as a tertiary care education and research hospital in Istanbul, Türkiye between August 1, 2025, and November 1, 2025, with a total of 130 participants. The study population consisted of 150 pediatricians (including residents, specialists, and faculty members) currently working at the institution. To determine the required sample size, a power analysis was performed using G*Power 3.1. Based on a 95% confidence level and a 5% margin of error, a minimum of 108 participants was targeted. A total of 130 pediatricians successfully completed the survey, resulting in a 86.6% response rate. The study was conducted in accordance with the Declaration of Helsinki. Approval for the study was obtained from the Non-Interventional Research Ethics Committee of Prof. Dr. Cemil Taşcıoğlu City Hospital, with decision number 370. The study employed a convenience sampling method, inviting all pediatric residents and specialists actively working in the hospital during the study period to participate voluntarily.

### Participants

#### Inclusion and exclusion criteria

Inclusion Criteria:

- Licensed pediatricians, including pediatric residents, specialists, and sub-specialists, currently employed at Prof. Dr. Cemil Taşcıoğlu City Hospital.
- Physicians who volunteered to participate in the study and provided written informed consent.

Exclusion Criteria:

- Healthcare professionals working in departments other than pediatrics.
- Pediatricians who were on long-term leave (e.g., medical leave, maternity leave) or not actively involved in clinical practice during the data collection period.
- Participants who submitted incomplete survey forms or withdrew their consent during the data collection process.

### Data collection tools

In addition to the participants’ sociodemographic characteristics, the general attitude scale towards artificial intelligence and the medical artificial intelligence readiness scale were administered.

#### Sociodemographic characteristics

It consists of questions (Gender, Age, Branch Title, Academic Title, Professional Experience).

#### Medical Artificial Intelligence Readiness Scale for Medical Students (MAIRS-MS)

It consists of 22 questions. The questions are gathered in 4 groups. The questions were prepared using a 5-point Likert scale, with 1 = strongly disagree and 5 = strongly agree. Scoring is done as follows: Cognition Factor (Items 1–8 Min: 8 Max: 40 points), Ability Factor (Items 9–16 Min: 8 Max: 40 points), Vision Factor (Items 17–19 Min: 3 Max: 15 points), Ethics Factor (Items 20–22 Min: 3 Max: 15 points), Medical Artificial Intelligence Readiness (MAIRS) total score (Items 1–22 Min: 22 Max: 110 points). The scale was developed, and the Turkish validity and reliability study was conducted by Karaca et al. [1]. Although initially designed for medical students, the MAIRS-MS dimensions (Cognition, Ability, and Vision) are highly applicable to clinicians in practice. In this study, the scale demonstrated excellent internal consistency (Cronbach’s alpha = 0.972), confirming its reliability for the pediatrician population. The ‘Cognition Factor’ assesses participants’ theoretical understanding of AI in medicine, while the ‘Ability Factor’ reflects their perceived technical proficiency in using AI tools. The ‘Vision’ and ‘Ethics’ factors evaluate their perspective on future applications and moral considerations, respectively. The Medical Artificial Intelligence Readiness Scale for Medical Students (MAIRS-MS) was utilized in this study. Although originally developed for medical students, its applicability to specialist physicians has been previously demonstrated in the literature, notably by Mertoğlu et al. [15], who adapted and validated the scale for practicing clinicians. In our study, the scale’s reliability was further confirmed by an excellent internal consistency (Cronbach’s alpha = 0.972), supporting its use in assessing AI readiness among pediatric specialists.

#### General Attitudes to Artificial Intelligence Scale (GAAIS)

The survey consisted of 20 questions. It was prepared using a 5-point Likert-type scale, with responses ranging from 1. Strongly disagree with 5. Strongly agree. The Turkish validity and reliability study was conducted by Kaya et al. [3]. In this study, the scale demonstrated excellent internal consistency (Cronbach’s alpha = 0.929), confirming its reliability for the pediatrician population.

### Statistics

SPSS 27 (Statistical Package for the Social Sciences) was used to analyze the study’s findings. Quantitative variables were reported as mean ± standard deviation, median, minimum, and maximum, while qualitative variables were reported using descriptive statistics such as frequency and percentage. The Shapiro-Wilk test, skewness and kurtosis indices, and box plots were used to assess the data’s conformity to a normal distribution. Student t-tests were used for two-group quantitative analyses with normal distributions, and Paired Sample t-tests were used for within-group analyses. One-way ANOVA was used for comparisons among three or more groups, and the Bonferroni test was used to identify the group responsible for the difference. Kruskal-Wallis tests were used for comparisons of three or more groups with variables that did not show a normal distribution, and the Dunn test was used to identify the group that caused the difference. Pearson correlation analysis was performed to assess relationships among variables based on their distributions. Linear regression analysis was also conducted to investigate the effects of Descriptive and AI-related characteristics on medical AI readiness levels. Linear regression analyses were performed to identify potential predictors of higher AI attitude scores and higher medical AI readiness scores. Results were evaluated at a 95% confidence interval, and significance was at *p* < 0.05.

## Results

### Participant characteristics

The study was conducted at Prof. Dr. Cemil Taşcıoğlu City Hospital between August 1, 2025, and November 1, 2025, with a total of 130 participants, 58.5% (*n* = 76) female and 41.5% (*n* = 54) male. The participants’ ages ranged from 25 to 65, with an average of 37.47 ± 9.31 years. According to specialty and title distribution, 22.3% (*n* = 29) of participants were pediatric subspecialists, 41.5% (*n* = 54) were pediatric residents, and 36.2% (*n* = 47) were general pediatric specialists. In terms of academic titles, 43.1% (*n* = 56) of the participants had no title, 46.2% (*n* = 60) were research assistants, 1.5% (*n* = 2) were assistant professors, 6.9% (*n* = 9) were associate professors, and 2.3% (*n* = 3) were professors. When the duration of duty was examined, 46.9% (*n* = 61) of the participants had been on duty for 1–5 years, 13.1% (*n* = 17) for 6–10 years, 13.8% (*n* = 18) for 11–15 years, 8.5% (*n* = 11) for 16–20 years, and 17.7% (*n* = 23) for 21 years or more (Table 1).

**Table 1:** Distrubution of descriptive statistics and examining the internal consistency of the GAAIS and MAIRS-MS scores

		*n* (%)
Gender	Female	76 (58,5)
	Male	54 (41,5)
Age	Mean ± Sd	37,47 ± 9,31
	Median (Min-Max)	35,5 (25–65)
Branch Title	Pediatric subspecialist	29 (22,3)
	Pediatric assistant	54 (41,5)
	General pediatrician	47 (36,2)
Academic Title	None	56 (43,1)
	Research assistant	60 (46,2)
	Assistant Professor	2 (1,5)
	Associated Professor	9 (6,9)
	Professor	3 (2,3)
Professional Experience	1–5 years	61 (46,9)
	6–10 years	17 (13,1)
	11–15 years	18 (13,8)
	16–20 years	11 (8,5)
	21 years and over	23 (17,7)

### AI attitudes and readiness scores

The scores that the participants received from the AI Positive Attitude sub-dimension ranged from 1 to 5, with an average of 3.81 ± 0.74 points; and the scores they received from the AI Negative Attitude sub-dimension ranged from 1 to 5, with an average of 2.83 ± 0.81 points. When the MAIRS-MS sub-dimension scores are examined; the “Cognition Factor” sub-dimension scores vary between 8 and 40, with an average of 26.88 ± 6.58 points; the “Ability Factor” sub-dimension scores vary between 8 and 40, with an average of 25.37 ± 5.31 points; the “Vision Factor” sub-dimension scores vary between 3 and 15, with an average of 11.11 ± 2.73 points; the “Ethics Factor” sub-dimension scores vary between 3 and 15, with an average of 11.61 ± 2.74 points; and the total MAIRS-MS scores range between 22 and 110, with an average of 74.97 ± 13.01 points (Table 2).

**Table 2:** Comparison of MAIRS-MS and GAAIS scores based on descriptive characteristics

			Cognition Factor	Ability Factor	Vision Factor	Ethics Factor	Total Point	AI Positive Attitude	AI Negative Attitude
			Mean ± Sd	Mean ± Sd	Mean ± Sd	Mean ± Sd	Mean ± Sd	Mean ± Sd	Mean ± Sd
Gender	Male		27,35 ± 7,33	25,46 ± 5,54	11,24 ± 2,98	11,76 ± 2,86	75,81 ± 14,99	3,8 ± 0,68	2,83 ± 0,82
	Female		26,55 ± 6,01	25,3 ± 5,18	11,01 ± 2,55	11,5 ± 2,67	74,37 ± 11,46	3,84 ± 0,82	2,84 ± 0,82
		^**a**^ **p**	***0***,***511***	***0***,***866***	***0***,***642***	***0***,***597***	***0***,***553***	***0***,***778***	***0***,***951***
Age	R		-0,123	-0,116	-0,212	-0,205	-0,197	-0,235	0,022
	P		***0***,***163***	***0***,***188***	***0***,016*	***0***,019*	***0***,024*	***0***,007**	***0***,***804***
Branch Title	Pediatric Sub-specialist		27,45 ± 5,92	25,59 ± 4,47	11,21 ± 2,77	11,83 ± 2,3	76,07 ± 11,72	3,78 ± 0,61	2,96 ± 0,61
	Pediatric Assistant		27,07 ± 6,07	25,80 ± 5,37	11,41 ± 2,4	11,89 ± 2,57	76,17 ± 9,89	3,94 ± 0,58	2,84 ± 0,86
	General Pediatrician		26,32 ± 7,54	24,74 ± 5,76	10,70 ± 3,06	11,15 ± 3,15	72,91 ± 16,47	3,7 ± 0,93	2,75 ± 0,88
		^**b**^ **p**	***0***,***742***	***0***,***596***	***0***,***425***	***0***,***358***	***0***,***402***	***0***,***258***	***0***,***541***
Academic Title	None		27,09 ± 7,45	25,41 ± 5,71	10,91 ± 3,13	11,38 ± 3,07	74,79 ± 16,12	3,76 ± 0,87	2,77 ± 0,87
	Research Assistant		26,77 ± 6,03	25,77 ± 5,17	11,18 ± 2,43	11,8 ± 2,5	75,52 ± 10,06	3,90 ± 0,61	2,86 ± 0,83
	Professor (Assist.,Assoc. Etc)		26,57 ± 5,39	23,50 ± 4,09	11,57 ± 2,34	11,71 ± 2,46	73,36 ± 10,75	3,65 ± 0,63	2,96 ± 0,48
		^**c**^ **p**	***0***,***752***	***0***,***071***	***0***,***780***	***0***,***878***	***0***,***516***	***0***,***361***	***0***,***778***
Professional Experience	1–5 years		27,38 ± 5,87	26,30 ± 5,48	11,41 ± 2,35	11,82 ± 2,53	76,90 ± 9,86	3,98 ± 0,58	2,89 ± 0,85
	6–10 years		26,82 ± 6,98	24,53 ± 4,78	10,76 ± 2,36	11,53 ± 2,21	73,65 ± 11,90	3,71 ± 0,79	2,70 ± 0,82
	11–15 years		27,67 ± 5,89	25,50 ± 3,13	11,78 ± 3,21	12,22 ± 2,80	77,17 ± 11,04	3,94 ± 0,61	2,65 ± 0,74
	16–20 years		29,27 ± 5,73	26,18 ± 3,43	11,64 ± 1,96	12,64 ± 1,69	79,73 ± 8,78	4,02 ± 0,50	2,92 ± 0,64
	21 years and over		23,87 ± 8,31	23,04 ± 6,72	9,78 ± 3,50	10,13 ± 3,53	66,83 ± 19,97	3,26 ± 0,98	2,88 ± 0,86
		^**c**^ **p**	***0***,***204***	***0***,***131***	***0***,***152***	***0***,***136***	***0***,019*	***0***,007**	***0***,***839***

^a^ Student t Test

r: Pearson’s Correlation Coefficient

^b^ One Way ANOVA Test

^c^ Kruskal Wallis Test&Dunn Bonferroni Test

**p < 0,01 *p < 0,05

*Sd* Standart Deviation

Statistical analysis revealed a significant negative correlation between age and several key variables. Specifically, as age increased, AI Positive Attitude scores (*r* = -0.235, *p* = 0.007, *p* < 0.01), Vision Factor scores (*r* = -0.212, *p* = 0.016, *p* < 0.05), and Ethics Factor scores (*r* = -0.205, *p* = 0.019) significantly decreased. Furthermore, a significant negative correlation was found between age and total MAIRS-MS scores (*r* = -0.197, *p* = 0.024, *p* < 0.05), indicating that younger pediatricians generally exhibit higher levels of AI readiness. No significant relationship was observed between age and AI Negative Attitude, Cognition Factor, or Ability Factor scores (*p* > 0.05) (Table 2).

Statistical analyses revealed no significant differences in AI Positive Attitude, AI Negative Attitude, or any of the MAIRS-MS sub-dimension and total scores based on gender, branch title, or academic title (*p* > 0.05). The distribution of readiness levels and attitudes remained statistically consistent across these demographic and professional categories (Table 2).

There is a statistically significant difference in AI Positive Attitude scores based on professional experience (*p* = 0.007; *p* < 0.01). Participants with 21 or more years of professional expertise had lower scores than those with 1–5, 11–15, and 16–20 years (*p* = 0.003; *p* = 0.010; *p* = 0.007; *p* < 0.05). Post-hoc analyses indicated that participants with 21 or more years of experience had significantly lower scores in both AI Positive Attitude and total MAIRS-MS compared to those with 1–5, 11–15, and 16–20 years of practice (*p* < 0.05). No statistically significant differences were observed in AI Negative Attitude or the individual MAIRS-MS sub-dimensions (Cognition, Ability, Vision, and Ethics) across experience levels (*p* > 0.05) (Table 2).

**Fig. 1:**
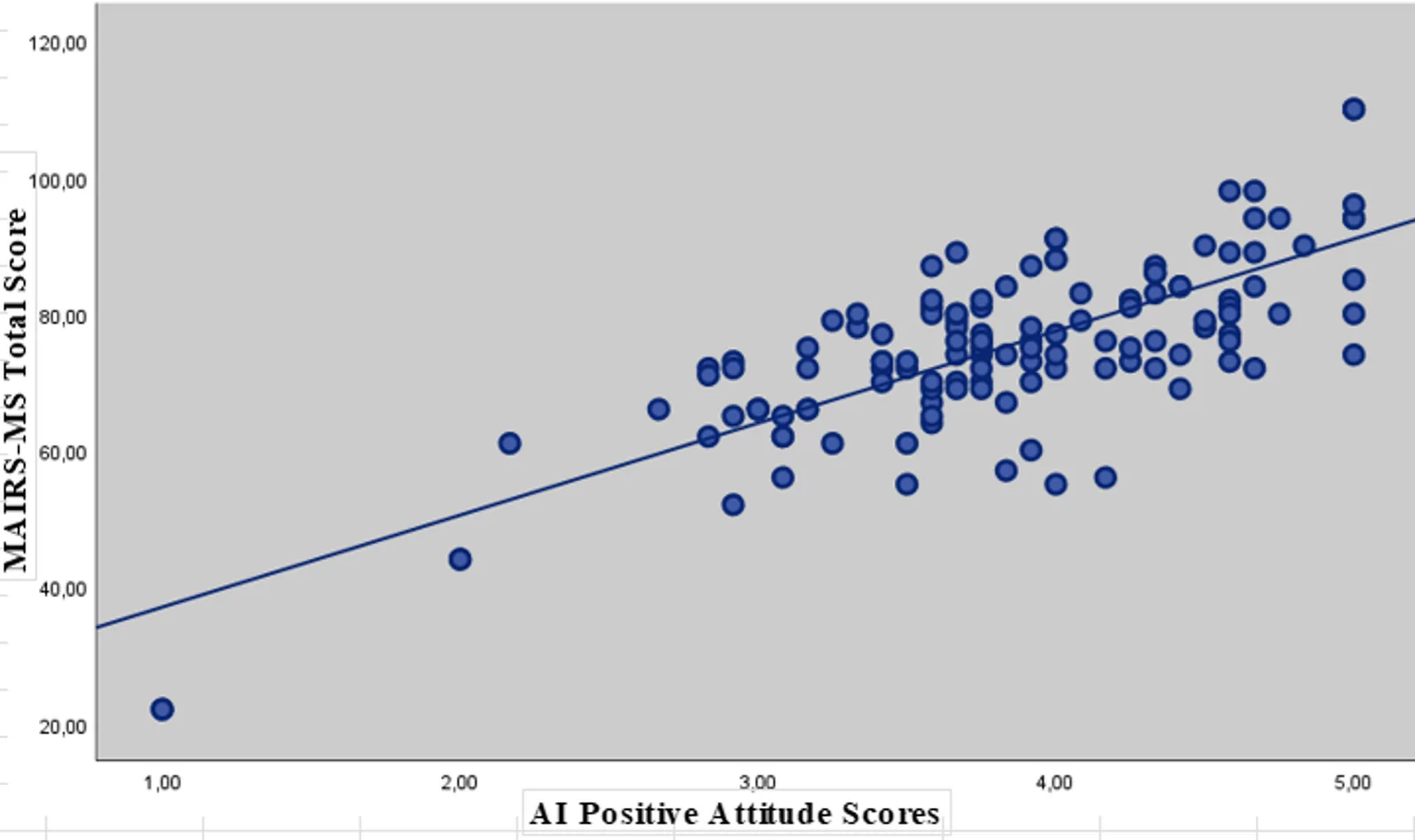
Relationship between MAIRS-MS total score and AI positive attitude score

A statistically significant positive correlation was found between the Cognition Factor (*r* = 0.603; *p* = 0.000; *p* < 0.01). A statistically significant positive correlation was found between the Ability Factor (*r* = 0.629; *p* = 0.000; *p* < 0.01). A statistically significant positive correlation was found between the Vision Factor (*r* = 0.497; *p* = 0.000; *p* < 0.01). A statistically significant positive correlation was found between the Ethics Factor (*r* = 0.460; *p* = 0.000; *p* < 0.01). A statistically significant positive correlation was found between the MAIRS-MS Total Score and AI Positive Attitude (*r* = 0.764; *p* = 0.000; *p* < 0.01) (Fig. 1).

No statistically significant correlation was found between the Cognition Factor (*p* = 0.315; *p* > 0.05). A statistically significant positive correlation was found between the Ability Factor (*r* = 0.762; *p* = 0.000; *p* < 0.01). A statistically significant positive correlation was found between the MAIRS-MS Total Score and AI Negative Attitudes (*r* = 0.228; *p* = 0.009; *p* < 0.01).

### Correlation and regression analyses

A multiple linear regression analysis was performed to identify the factors influencing pediatricians’ AI-positive attitudes. The model was statistically significant (F = 39.382; *p* = 0.001), accounting for 65.8% of the variance in positive attitude levels (R^2^ = 0.658). The Ability factor (β = 0.516, *p* < 0.001) and Cognition factor (β = 0.323, *p* < 0.001) were found to be the strongest predictors of a positive attitude. This indicates that as pediatricians’ perceived technical competence and conceptual knowledge increase, their positive outlook toward AI technologies significantly improves (Table 3).

**Table 3:** Linear regression analysis results

Dependent Variable		Independent Variable		ß	t		*p*		F	Model (*p*)	*R* ^2^
AI Positive Attitude		Coefficient			1,171		**0**,**244**		39,382	**0**,001**	0,658
		**Age**		0,044	0,373		**0**,**709**				
		**Professional experience**		-0,151	-1,274		**0**,**205**				
		**Cognition Factor**		0,323	4,153		**0**,001**				
		**Ability Factor**		0,516	9,288		**0**,001**				
		**Vision Factor**		0,194	1,744		**0**,**084**				
		**Ethics Factor**		0,021	0,199		**0**,**843**				
Medical Artificial Intelligence Readiness Level		Coefficient			4,420		0,001**		59,641	0,001**	0,587
		**Age**		-0,131	-1,040		**0,300**				
		**Professional experience**		0,128	1,007		**0,316**				
		**AI Positive Attitude**		0,769	12,875		0,001**				

In the multivariate model, age, professional experience, vision, and ethical factors did not reach statistical significance as independent predictors of positive attitude (*p* > 0.05). A separate multiple regression analysis was conducted to evaluate the determinants of MAIRS-MS. The model proved to be statistically significant (F = 59.641; *p* = 0.001), explaining 58.7% of the variation in readiness levels (*R* = 0.587). AI Positive attitude was identified as the primary and most powerful predictor of MAIRS-MS (β = 0.769, *p* < 0.001). This suggests that a general favorable disposition toward AI is the most critical driver for a pediatrician’s readiness to implement these technologies in clinical practice (Table 3).

Age and professional experience were not found to be significant predictors within the multivariate readiness model (*p* > 0.300). This finding implies that the influence of age observed in univariate comparisons is largely mediated or overshadowed by the physician’s overall attitude toward the technology.

## Discussion

This study assessed pediatricians’ attitudes toward artificial intelligence and their preparedness to implement AI-based technologies in clinical practice. Our results showed that pediatricians generally have positive attitudes and moderate-to-high levels of AI readiness. However, advanced age and more years of professional experience were linked to lower readiness and more cautious perspectives on AI.

Our findings highlight that while pediatricians possess high ‘Vision’ for AI, a significant ‘Ability’ gap remains, particularly among senior clinicians. To ensure workforce preparedness, it is essential to integrate AI ethics and technical literacy into pediatric residency programs and develop targeted upskilling initiatives for experienced physicians. Clinically, AI should be implemented as a decision-support tool to transition pediatric care from a reactive to a proactive model, ensuring that technological integration enhances rather than replaces the essential human element of pediatric practice.

The correlation analysis revealed a significant negative relationship between advanced age and professional experience and AI readiness, indicating that resistance or a cautious attitude towards technological adaptation develops as professional seniority increases. On the other hand, the results of the multiple linear regression analysis reveal the underlying mechanisms of this attitude; Cognition, Ability, and Positive Attitude were found to be the strongest and most significant predictors of AI readiness. This suggests that the low level of readiness among senior physicians is not merely an age-related preference, but also stems from a perceived lack of technical skill and cognitive familiarity within this group.

Karaca et al. studied 897 medical students at two state universities in Türkiye and found the MAIRS-MS Cronbach’s alpha reliability coefficient to be 0.87. The results demonstrated the concordance and construct validity of the four-factor model used in the scale [1]. Internal consistency was calculated as α = 0.916 for the “Cognition Factor,” α = 0.974 for the “Ability Factor,” α = 0.912 for the “Vision Factor,” α = 0.926 for the “Ethics Factor,” and α = 0.972 for the total scale in our study. Therefore, the Medical Artificial Intelligence Readiness Scale and all its subscales demonstrate a very high level of internal consistency. Specifically, the Cronbach’s Alpha coefficient for the total scale reflects a high level of reliability.

In the study conducted by Kaya et al. with 360 individuals aged 18–51 using the GAAIS, it was noted that personality traits, artificial intelligence anxiety, and demographic characteristics significantly influence attitudes toward artificial intelligence. The Cronbach’s α values were 0.82 for AI positive attitude and 0.84 for AI negative attitude [2]. When examining internal consistencies, the Cronbach’s alpha coefficient was calculated as α = 0.929 for the AI Positive Attitude sub-dimension and α = 0.895 for the AI Negative Attitude sub-dimension. These values indicate that the GAAIS has high reliability.

National and institutional ethical frameworks in Türkiye emphasize the importance of human oversight and medico-legal accountability in the use of artificial intelligence in healthcare. The moderate AI readiness scores observed in this study may reflect pediatricians’ hesitancy to fully integrate AI tools into routine practice in the absence of clearly defined legal responsibilities. Although strategic documents highlight principles such as patient safety, data privacy, and the “do no harm” approach, the lack of specific medico-legal regulations addressing AI-related clinical errors remains a challenge for clinicians [12–14].

The cautious optimism identified among pediatricians in this study appears to be consistent with these ethical frameworks, which promote careful and supervised implementation of AI rather than rapid adoption. The absence of locally applicable clinical guidelines may place pediatricians in a position of ethical uncertainty, potentially contributing to the observed discrepancy between relatively high “Vision” scores and lower confidence in “Ability.” This gap suggests that while clinicians recognize the potential value of AI, uncertainty regarding accountability and practical implementation may limit their readiness to use these tools independently.

Furthermore, the lower readiness levels observed among more experienced pediatricians may be partly explained by heightened awareness of medico-legal responsibility and data governance issues. In the current regulatory context, senior clinicians may adopt a more conservative stance when clinical liability related to AI-supported decision-making is not clearly delineated [12–14]. This finding underscores the importance of aligning ethical principles with practical guidance that supports clinicians in daily practice.

Taken together, these findings suggest that improving AI readiness among pediatricians may require not only educational initiatives but also clearer institutional and regulatory frameworks. Structured training programs focusing on AI interpretability, ethical oversight, and clinician-centered use may help translate positive perceptions into practical confidence, particularly when supported by clearly defined legal and governance structures consistent with national AI strategies [12–14].

In the study by Mertoğlu et al., no significant differences in physicians’ readiness for medical AI were found by age, gender, or marital status; only professional experience had a substantial effect on the vision subscale [15]. However, in our study, age was negatively correlated with both AI-positive attitude and total MAIRS-MS scores. In addition, physicians with 21 or more years of professional experience had significantly lower AI readiness and positive attitude scores. These differences may be attributed to variations in study populations, institutional technological infrastructure, and exposure to digital transformation processes.

Denizli et al. reported that medical students generally had high levels of AI readiness but relatively low scores on the cognition subscale [16]. In our study, cognition scores were comparatively higher. This difference may be explained by the effect of clinical experience, which may increase not only practical exposure but also conceptual understanding of AI applications. While Denizli et al. found significant effects of demographic variables on AI readiness, our study found no significant differences in AI readiness by gender, academic title, or medical branch.

In a study conducted by Hamad et al. among medical students in Jordan, the average AI readiness score was reported as 64.2%, and academic performance was positively correlated with AI readiness [17]. In our study, the mean total MAIRS-MS score was 74.97 ± 13.01, which is higher than that reported in medical students. This could be due to the greater exposure of practicing pediatricians to clinical technologies and decision support systems in real-world practice.

In a study conducted in India with 482 undergraduate medical students, the mean MAIRS-MS score was reported as 74.61 ± 10.137 [18], which closely aligns with the mean score in our study (74.97 ± 13.01). However, the authors highlighted significant variability among individuals, especially in cognition and ability domains. Similarly, in our study, participants with greater age and more professional experience had lower scores, suggesting that generational differences may affect AI adaptation.

In a study among participants in the International Cooperation and Exchange Program (ICEP), 41.82% agreed or strongly agreed that AI education should be included in the medical curriculum, and overall AI readiness was positively associated with exposure to AI training [19]. Similarly, our study showed a strong positive correlation between a positive attitude towards AI and AI readiness (*r* = 0.764). These findings indicate that incorporating AI education into medical training programs may significantly enhance both attitudes and readiness.

In the study conducted in Saudi Arabia, higher AI readiness scores were observed among female students, those living in central regions, and those with advanced English and computer skills [20]. In contrast, gender had no significant effect on AI readiness in our study, while age and professional experience were significant predictors. This difference may result from variations between student populations and practicing physicians, as well as differences in educational backgrounds and institutional settings.

In a study by Perrier et al., involving 165 pediatricians in France, most participants indicated that AI would improve healthcare, and 86% supported implementing AI tools in pediatrics. Although 59% of participants viewed AI as a threat to medical data security, 35% perceived it as a threat to the ethical and human aspects of medicine. Only 5% of participants had received specialized AI training, but those who did exhibited significantly higher knowledge levels and were more likely to use AI tools in their medical practices [21]. In our study, pediatricians generally showed positive attitudes toward AI, as reflected in their relatively high scores on positive attitude measures, while also expressing concerns through their negative attitude scores.

In a study of ChatGPT’s use in a pediatric intensive care unit, healthcare professionals appreciated its user-friendliness and potential to accelerate tasks and deliver quick information. Still, they expressed concerns about data reliability, timeliness, and ethical issues. They also showed cautious optimism about AI [22]. This matches our findings, in which pediatricians displayed moderate-to-high readiness but did not score very high across all subdimensions, especially in the ethics and vision domains, when analyzed by age. Additionally, the negative correlation we found between age and both positive attitudes toward AI and the overall MAIRS-MS score suggests that more experienced clinicians may be more cautious about AI tools, as intensive care staff in this study expressed similar concerns.

In a study of 20 caregivers and 22 clinicians using 11 different AIs, participants expressed concerns about AI’s accuracy, privacy, and the potential loss of empathy. Still, they believed it could provide real-time document translation and more comfortable verbal communication [23]. The strong positive correlation between favorable attitudes toward AI and overall readiness in our study indicates that pediatricians are more inclined to accept its integration into pediatric care when they view AI as supportive rather than threatening.

A cross-sectional survey of primary care physicians in Switzerland revealed a significant knowledge-attitude gap among participants. While 69.0% of participants held a positive attitude towards AI and 81.9% sought training opportunities, only 14.8% rated their knowledge as high or excellent. Despite the acknowledged competency gap, 27.7% of physicians already use AI tools for clinical purposes [24]. Unlike the Swiss primary care setting, our focus on a tertiary hospital in Istanbul revealed slightly higher overall readiness, potentially due to greater exposure to advanced digital decision-support systems. Nevertheless, both studies converge on the same conclusion: there is an urgent global need for structured medical AI curricula to bridge the gap between optimistic attitudes and safe, competent clinical application.

Heinrichs et al. study, Physicians reported high AI enthusiasm and lower skepticism; reverse coded, with higher scores indicating reduced skepticism). Greater AI familiarity, use in daily life or professionally, and involvement in research were strongly associated with greater enthusiasm and lower skepticism. Chief physicians were significantly less skeptical than residents; however, age and discipline did not influence attitudes [25]. While Heinrichs et al. found that age did not influence attitudes and that chief physicians were less skeptical than residents, our study revealed a significant negative correlation between advanced age and AI readiness.

In recent study by Serin et al. [26], it was found that 73.6% of Turkish physicians (pediatricians and family physicians) caring for children have limited knowledge of artificial intelligence, despite a high interest in advanced training, and only 8.3% trust fully autonomous AI, with the majority (64.5%) placing the burden of accuracy and accountability on algorithm designers rather than clinicians. They noted that the digital divide in the context of Turkish healthcare is not only age-related but also related to a lack of institutional support and policy. Similar to our cohort’s high ‘Vision’ but low ‘Ability’ scores, their study highlights a ‘knowledge-trust gap’ where physicians recognize the potential of AI but hesitate due to a lack of formal training and technical competence.

Akyon et al. [27] who reported a median readiness score of 62.7% among young European family doctors. The slightly higher readiness(%68) in our cohort may stem from the advanced technological infrastructure of Turkish tertiary hospitals compared to general primary care. Despite this, both studies confirm that AI readiness is primarily driven by knowledge of current applications, highlighting a global need for formal training. Notably, while Akyon et al. focused on ‘digital native’ young doctors, our study fills a literature gap by identifying advanced age as a negative predictor. This underscores that specialized ‘upskilling’ programs are urgent for senior clinicians to bridge the emerging digital divide within the pediatric workforce.

The ‘vision readiness gap’ shared in the study by Barile et al. [28] suggests that the potential of Large Language Models (LLMs) to provide real-time feedback and enhance bedside training is widely recognized by the pediatric health community. Our findings demonstrate a significant difference between high Vision scores and relatively low Ability scores among pediatricians. Pediatricians in our study group clearly perceive the transformative power of generative AI in medical education, from individualized study plans to the creation of complex clinical simulations. However, as Barile et al. emphasize, it is essential that these tools be used wisely by content experts to mitigate risks such as clinical ‘hallucinations’ and ethical biases. The high vision scores in our study reflect a workforce that is conceptually ready for this transformation but requires structured, formal training to ensure the transition to practical, safe clinical practice. The capacity of LLMs to process vast datasets makes them highly suitable for algorithmic problem solving.

While AI correctly often outperforming general practitioners in differential diagnosis, diagnosed 39% of complex NEJM pediatric case challenges its performance in pediatrics reveals a significant ‘accuracy gap.’ Recent benchmarks specifically in pediatrics show that LLMs struggle more with age-dependent variables and developmental nuances than with adult-centric cases [29]. This suggests that while LLMs are powerful complementary tools, their diagnostic accuracy is currently less reliable in the pediatric subspecialties, justifying the caution observed in our participants’ ‘Ability’ scores.

The caution observed among clinicians of advanced age in our study aligns with the ethical frameworks proposed by Boch et al. [30]. They emphasize that in pediatrics, the ‘do no harm’ principle must be extended to data privacy and the prevention of algorithmic bias. Senior pediatricians, who often act as the primary guardians of patient safety, may perceive the current lack of transparent and ethical AI guidelines as a significant barrier to implementation.

Mahajan et al. study taht further clarify this with their argument medical AI has a uniquely non-static nature. Unlike traditional medical technologies, AI models evolve through continuous data accumulation and updates, creating a risk of ‘performance drift’ that might go unnoticed without expert oversight [31]. This dynamic complexity justifies the caution observed among our participants, particularly senior pediatricians who, as primary guardians of patient safety, might find themselves not yet equipped to assess the reliability of evolving algorithms.

A critical finding of our study is the significant negative correlation between professional seniority and AI readiness. While simple lack of exposure is a factor, our results suggest a more complex interplay of institutional culture, training history, and clinical responsibility.

Senior pediatricians (those with ≥ 21 years of experience) were trained in a pre-digital era where clinical intuition was primarily built on physical examination and traditional diagnostic heuristics. For these clinicians, AI may not be seen as a simple “tool” but as a potential disruption to the established doctor-patient relationship and clinical autonomy. Furthermore, as senior specialists often carry the highest medico-legal and ethical responsibility within a tertiary hospital, their lower “Ability” scores (β = 0.516) likely reflect a principled caution. In the current “regulatory vacuum” regarding AI-driven errors in Türkiye, senior clinicians may perceive the lack of transparent, explainable AI (XAI) as a risk to patient safety rather than a resistance to change [30, 31].

Instead of viewing this as “technology resistance,” it should be interpreted as a knowledge-trust gap. Our regression analysis confirms this: Cognition and Ability were the strongest predictors of readiness. This indicates that senior physicians are not inherently opposed to AI, but their readiness is contingent upon receiving structured, evidence-based training that addresses the specific ethical and practical nuances of pediatric care, rather than general digital literacy.

The cautious attitude observed among pediatricians in our study reflects a multifaceted ‘clinical and functional anxiety.’ As evidenced by our regression analysis, where the Ability factor emerged as the most significant predictor of AI positive attitudes, the primary concern for clinicians is not the technology itself but rather a perceived lack of technical competence to handle AI safely in a high-stakes specialty like pediatrics. This functional anxiety is further intensified by the medico-legal uncertainties, as physicians fear being left in a regulatory vacuum regarding liability in the event of an algorithmic error. Furthermore, the negative correlation between advanced age and readiness suggests a deeper concern regarding the digital divide, where seasoned clinicians may feel their extensive clinical experience is being marginalized by emerging algorithmic models. Ultimately, the state of concern identified in our study is not a rejection of innovation, but a professional caution rooted in the absence of structured training, legal clarity, and the unique vulnerability of the pediatric patient population.

### Limitations of the study

This study has limitations in terms of generalizability of the results to primary healthcare services or institutions with different technological infrastructures, as it was conducted in a single tertiary center in Istanbul. Collecting data through self-report questionnaires carries a risk of response bias due to participants’ tendency to appear more positive towards innovations (social desirability bias); and the cross-sectional design prevents tracking changes in attitudes over time in a rapidly evolving field like artificial intelligence. Additionally, MAIRS-MS was originally created for medical students rather than practicing physicians. Despite these limitations, the study offers useful preliminary data and highlights the need for future multicenter and longitudinal research. This is a single-center study conducted in a tertiary academic hospital in Istanbul. The findings may not be fully generalizable to pediatricians working in primary care or different geographic regions with varying technological infrastructures. Artificial intelligence is a rapidly evolving field. These results provide a temporal snapshot; however, clinician attitudes and readiness levels may shift quickly as new technologies (e.g., LLMs) and specialized pediatric AI tools emerge. The study relies on self-reported data, which may be subject to social desirability bias. Additionally, although the MAIRS-MS scale is highly reliable, it may not fully capture the specific medico-legal and liability concerns of senior specialists.

## Conclusion

This study shows that pediatricians generally have positive attitudes and moderate-to-high readiness toward artificial intelligence at tertiary hospital in Istanbul. AI-positive attitudes are strongly linked to higher levels of readiness. However, advanced age and longer professional experience are associated with lower AI readiness and attitudes. No differences were observed based on gender, specialty, or academic title. These findings emphasize the need for structured AI education, especially for senior pediatricians, to promote the safe, effective, and ethical integration of AI into pediatric practice.

## Supplementary Information

Supplementary Material 1

Supplementary Material 2

Supplementary Material 3

## Data Availability

Available from the corresponding author on reasonable request.

## Abbreviations

AI: Artificial Intelligence
MAIRS-MS: Medical Artificial Intelligence Readiness Scale for Medical Students
GAAIS: General Attitudes toward Artificial Intelligence Scale
SD: Standard Deviation
SPSS: Statistical Package for the Social Sciences
ANOVA: Analysis of Variance
