# Supplementary material for: Assessing pediatricians’ readiness for artificial intelligence: a cross-sectional study in Istanbul, Türki̇ye
Source: BMC Pediatr. 2026 Jun 3;26:835. doi: 10.1186/s12887-026-07088-8 (PMC13548471; doi:10.1186/s12887-026-07088-8)
Supplement: Supplementary file 1 [file 12887_2026_7088_MOESM1_ESM.pdf]

2025 AİLE YILI

T.C.
İSTANBUL VALİLİĞİ
İL SAĞLIK MÜDÜRLÜĞÜ
İstanbul Prof. Dr. Cemil Taşcıoğlu Şehir Hastanesi

Sayı : E-48670771-514.99-292100675 21.10.2025
Konu : Etik Kurul (Eren Güzeloğlu)

Sayın Uzm. Dr. Eren GÜZELOĞLU
Çocuk Sağlığı ve Hastalıkları Kliniği

Kliniğinizde yürütmeyi planladığınız 'Çocuk Sağlığı ve Hastalıkları Hekimlerinin Yapay Zeka Genel Tutumunun ve Hazır Bulunuşluluğunun Değerlendirilmesi' isimli anket çalışmanız İstanbul Prof. Dr. Cemil Taşcıoğlu Şehir Hastanesi Bilimsel Araştırmalar Etik Kurulu'nun 20.10.2025 tarihli toplantısında incelenmiş, 370 sayılı karara göre etik açıdan uygun bulunmuştur.

Gereğini bilgilerinize rica ederim.

Doç. Dr. Müge ÖNER TAMAM
Etik Kurul Başkanı

Ek: Karar evrakı

GÜVENLİ ELEKTRONİK İMZALI ASLI İLE AYNIDIR

Bu belge, güvenli elektronik imza ile imzalanmıştır.
Belge doğrulama kodu: 964409C8-EFF7-431D-A954-B5E644AF57FE Belge doğrulama adresi: https://www.turkiye.gov.tr/saglik-bakanligi-ebys

Kaptan Paşa Mah. Darülaceze Cad. No:27 34384 Şişli/İstanbul 34384
Telefon No: 0212 314 55 55
e-Posta: İnternet Adresi: https://cemiltascioglush.saglik.gov.tr/
Kep Adresi: cemiltascioglusehirhastanesi@hs01.kep.tr

Bilgi için: Meryem Cansu OLT
Hemşire
Telefon No: 02122217777

CamScanner ile tarandı

İSTANBUL PROF. DR. CEMİL TAŞCIOĞLU ŞEHİR HASTANESİ GİRİŞİMSEL OLMAYAN KLİNİK ARAŞTIRMALAR ETİK KURULU KARAR FORMU

ÇALIŞMANIN ADI	Çocuk Sağlığı ve Hastalıkları Hekimlerinin Yapay Zeka Genel Tutumunun ve Hazır Bulunuşluluğunun Değerlendirilmesi
PROTOKOL KODU	370

ETİK KURUL BİLGİLERİ	ETİK KURULUN ADI	İSTANBUL PROF. DR. CEMİL TAŞCIOĞLU ŞEHİR HASTANESİ BİLİMSEL ARAŞTIRMALAR ETİK KURULU
	AÇIK ADRESİ:	Darülaceze Cad. No:25 Şişli/İSTANBUL
	TELEFON	0(212)314 55 88
	E-POSTA	cemiltascioglu.gokaetikkurul@gmail.com
	TOPLANTI TARİHİ	20.10.2025

Unvanı/Adı/Soyadı	Uzmanlık Alanı	Görevi	İmza
Doç. Dr. Müge ÖNER TAMAM	NÜKLEER TIP	BAŞKAN	
Doç. Dr. Uğur KESİCİ	GENEL CERRAHİ	BAŞKAN YARD.	
Prof. Dr. Murat ÇAKAR	ORTOPEDİ VE TRAVMATOLOJİ	ÜYE	
Prof. Dr. Vefik ARICA	ÇOCUK SAĞLIĞI VE HASTALIKLARI	ÜYE	
Doç. Dr. Mehmet Gökhan ÇULHA	ÜROLOJİ	ÜYE	
Doç. Dr. Yasemin GÖKDEN	İÇ HASTALIKLARI	ÜYE	
Doç. Dr. Belkız ÖNGEN İPEK	TIBBİ BİYOKİMYA	ÜYE	
Uzm. Dr. Aybüke BACANLI	AİLE HEKİMLİĞİ	ÜYE	
Uzm. Dr. Belen ATEŞ	FARMAKOLOJİ	ÜYE	
Uzm. Dr. Suzan ÖNOL	RADYOLOJİ	ÜYE	

Uzm. Dr. Burak Dyrok   Fizyoloji   Üye

CamScanner ile tarandı
