# Supplementary material for: Assessing pediatricians’ readiness for artificial intelligence: a cross-sectional study in Istanbul, Türki̇ye
Source: BMC Pediatr. 2026 Jun 3;26:835. doi: 10.1186/s12887-026-07088-8 (PMC13548471; doi:10.1186/s12887-026-07088-8)
Supplement: Supplementary file 2 [file 12887_2026_7088_MOESM2_ESM.docx]

### **Medical Artificial Intelligence Readiness Scale for Medical Students (MAIRS-MS)**

| 1-Strongly Disagree | 2-Disagree | 3-Neutral | 4-Agree | 5-Strongly Agree |
| --- | --- | --- | --- | --- |

1. I can define the basic concepts of data science

2. I can define the basic concepts of statistics

3. I can explain how AI systems are trained

4. I can define the basic concepts and terminology of AI

5. I can properly analyze the data obtained by AI in healthcare.

6. I can differentiate the functions and features of AI related tools and applications.

7. I can organize workflows compatible with AI.

8. I can express the importance of data collection, analysis, evaluation and safety; for the development of AI in healthcare.

9. I can harness AI-based information combined with my professional knowledge.

10. I can use AI technologies effectively and efficiently in healthcare delivery.

11. I can use artificial intelligence applications in accordance with its purpose.

12. I can access, evaluate, use, share and create new knowledge using information and communication technologies.

13. I can explain how AI applications offer a solution to which problem in healthcare.

14. I find valuable to use AI for education, service and research purposes.

15. I can explain the AI applications used in healthcare services to the patient.

16. I can choose proper AI application for the problem encountered in healthcare.

17. I can explain the limitations of AI technology.

18. I can explain the strengths and weaknesses of AI technology.

19. I can foresee the opportunities and threats that AI technology can create.

20. I can use health data in accordance with legal and ethical norms.

21. I can conduct under ethical principles while using AI technologies.

22. I can follow legal regulations regarding the use of AI technologies in healthcare.

Cognition Factor: 1-8 Items Min:8 Max:40 points

Ability Factor: 9-16 Items Min:8 Max:40 points

Vision Factor: 17-19 Items Min:3 Max:15 points

Ethics Factor: 20-22 Items Min:3 Max:15 points

Medical Artificial Intelligence Readiness: 1-22 Items Min:22 Max:110 points
