# Supplementary material for: Assessing pediatricians’ readiness for artificial intelligence: a cross-sectional study in Istanbul, Türki̇ye
Source: BMC Pediatr. 2026 Jun 3;26:835. doi: 10.1186/s12887-026-07088-8 (PMC13548471; doi:10.1186/s12887-026-07088-8)
Supplement: Supplementary file 3 [file 12887_2026_7088_MOESM3_ESM.docx]

**Supplementary Material – Turkish Version of the General Attitudes to Artificial Intelligence Scale**

**Yapay Zekâya Yönelik Genel Tutum Ölçeği, Kaya *et al*.** (2022). <https://doi.org/10.1080/10447318.2022.2151730>

| Yönerge: Değerli katılımcı. Bu anket yapay zekaya yönelik tutumlarınız hakkında bilgi almak için tasarlanmıştır. Lütfen her bir maddeyi dikkatle okuyarak size en uygun olan seçeneği 1 ile 5 arasında işaretleyiniz. | Kesinlikle katılmıyorum | Katılmıyorum | Kararsızım | Katılıyorum | Kesinlikle katılıyorum |
| --- | --- | --- | --- | --- | --- |
| 1. Günlük hayatımda yapay zekâ sistemlerini kullanmak ilgimi çekiyor. | 1 | 2 | 3 | 4 | 5 |
| 2. Yapay zekânın birçok faydalı uygulaması vardır. | 1 | 2 | 3 | 4 | 5 |
| 3. Yapay zekâ heyecan vericidir. | 1 | 2 | 3 | 4 | 5 |
| 4. Yapay zekâ bu ülke için yeni ekonomik fırsatlar sağlayabilir. | 1 | 2 | 3 | 4 | 5 |
| 5. Yapay zekâyı kendi işimde kullanmak isterim. | 1 | 2 | 3 | 4 | 5 |
| 6. Yapay zekâya sahip bir yazılım/robot, birçok rutin işi bir insandan daha iyi yapabilir. | 1 | 2 | 3 | 4 | 5 |
| 7. Yapay zekânın yapabileceklerinden etkilendim. | 1 | 2 | 3 | 4 | 5 |
| 8. Yapay zekanın insanların iyi oluşları üzerinde olumlu etkileri olabilir. | 1 | 2 | 3 | 4 | 5 |
| 9. Yapay zekâlı sistemler insanların daha mutlu hissetmelerine yardımcı olabilir. | 1 | 2 | 3 | 4 | 5 |
| 10. Yapay zekâlı sistemler insanlardan daha iyi performans gösterebilir. | 1 | 2 | 3 | 4 | 5 |
| 11. Toplumun çoğu, yapay zekâ ile donatılmış bir gelecekten faydalanacaktır. | 1 | 2 | 3 | 4 | 5 |
| 12. Rutin işlemler için, bir insan yerine yapay zekâlı bir sistemle etkileşime girmeyi tercih ederim. | 1 | 2 | 3 | 4 | 5 |
| 13. Yapay zekânın tehlikeli olduğunu düşünüyorum. | 1 | 2 | 3 | 4 | 5 |
| 14. Kuruluşlar yapay zekâyı etik olmayan bir şekilde kullanırlar. | 1 | 2 | 3 | 4 | 5 |
| 15. Yapay zekâyı şeytani/kötü niyetli buluyorum. | 1 | 2 | 3 | 4 | 5 |
| 16. Yapay zekâ insanları gözetlemek için kullanılır. | 1 | 2 | 3 | 4 | 5 |
| 17. Yapay zekânın gelecekteki kullanımlarını düşündüğümde üzüntüden titriyorum. | 1 | 2 | 3 | 4 | 5 |
| 18. Yapay zekâ insanların kontrolünü ele geçirebilir. | 1 | 2 | 3 | 4 | 5 |
| 19. Yapay zekâlı sistemlerin birçok hata yaptığını düşünüyorum. | 1 | 2 | 3 | 4 | 5 |
| 20. Yapay zekâ gitgide daha fazla kullanılırsa benim gibi insanların zarar göreceğini düşünüyorum. | 1 | 2 | 3 | 4 | 5 |

Original English items, with item numbers matching the Turkish version above:

1. I am interested in using artificially intelligent systems in my daily life.
2. There are many beneficial applications of Artificial Intelligence.
3. Artificial Intelligence is exciting.
4. Artificial Intelligence can provide new economic opportunities for this country.
5. I would like to use Artificial Intelligence in my own job.
6. An artificially intelligent agent would be better than an employee in many routine jobs.
7. I am impressed by what Artificial Intelligence can do.
8. Artificial Intelligence can have positive impacts on people’s wellbeing.
9. Artificially intelligent systems can help people feel happier.
10. Artificially intelligent systems can perform better than humans.
11. Much of society will benefit from a future full of Artificial Intelligence.
12. For routine transactions, I would rather interact with an artificially intelligent system than with a human.
13. I think Artificial Intelligence is dangerous.
14. Organisations use Artificial Intelligence unethically.
15. I find Artificial Intelligence sinister.
16. Artificial Intelligence is used to spy on people.
17. I shiver with discomfort when I think about future uses of Artificial Intelligence.
18. Artificial Intelligence might take control of people.
19. I think artificially intelligent systems make many errors.
20. People like me will suffer if Artificial Intelligence is used more and more.

Note that a randomised order of these items, with full English instructions, and an attention check, can be found in Schepman & Rodway (2022), and please note that the item numbers differ in that item list compared to the list here. The order here reflects Schepman and Rodway (2020), Table 3. In the Turkish version, items 1-12 constitute the positive subscale, and items 13-20 the negative subscale. To score, reverse-score the negative items, and calculate separate overall means for the positive and negative items.

**References**

Kaya, F., Aydin, F., Schepman, A., Rodway, P., Yetişensoy, O., & Demir Kaya, M. (2022). The roles of personality traits, AI anxiety, and demographic factors in attitudes towards artificial intelligence. *International Journal of Human–Computer Interaction.* <https://doi.org/10.1080/10447318.2022.2151730>

Schepman, A. & Rodway, P. (2020). Initial validation of the general attitudes towards Artificial Intelligence Scale. *Computers in Human Behavior Reports* (1), 100014. <https://doi.org/10.1016/j.chbr.2020.100014>

Schepman, A. & Rodway, P. (2022). The General Attitudes towards Artificial Intelligence Scale (GAAIS): Confirmatory Validation and Associations with Personality, Corporate Distrust, and General Trust. *International Journal of Human–Computer Interaction* <https://doi.org/10.1080/10447318.2022.2085400>
